# Primary pericardial synovial sarcoma requiring emergency salvage right atrial debulking: a case report

**DOI:** 10.1093/jscr/rjae372

**Published:** 2024-06-03

**Authors:** Ramanish Ravishankar, Rishab Makam, Mahmoud Loubani, Mubarak Chaudhry, Azar Hussain

**Affiliations:** Faculty of Public Health, London School of Hygiene and Tropical Medicine, London WC1E 7HT, United Kingdom; Department of Cardiothoracic Surgery, CastleHill Hospital, Hull HU16 5JQ, United Kingdom; Department of Cardiothoracic Surgery, CastleHill Hospital, Hull HU16 5JQ, United Kingdom; Department of Cardiothoracic Surgery, CastleHill Hospital, Hull HU16 5JQ, United Kingdom; Department of Cardiothoracic Surgery, CastleHill Hospital, Hull HU16 5JQ, United Kingdom

**Keywords:** pericardial tumour, cardiac surgery

## Abstract

A 52-year-old gentleman presented with symptoms of breathlessness and type 1 respiratory failure. His CT pulmonary angiogram showed a heterogenous, oval-shaped lesion between the heart and diaphragm with a right atrial (RA) filling defect, pericardial thickening and pulmonary metastasis. An RA debulking salvage operation confirmed this to be a pericardial tumour and further cytology and immunohistochemistry testing confirmed a primary synovial sarcoma. After 12 days in intensive care for ventilation, the patient was successfully discharged on warfarin and underwent oncology follow-up for chemotherapy. Following a 15-month follow-up, no mortality was observed despite the aggressive nature of the tumour.

## Introduction

Primary pericardial synovial sarcoma is a rare pathology with an often, uncertain prognosis. This report presents a case of a gentleman with acute respiratory failure as a result of a pericardial synovial sarcoma requiring emergency salvage debulking.

## Case summary

**Figure 1:**
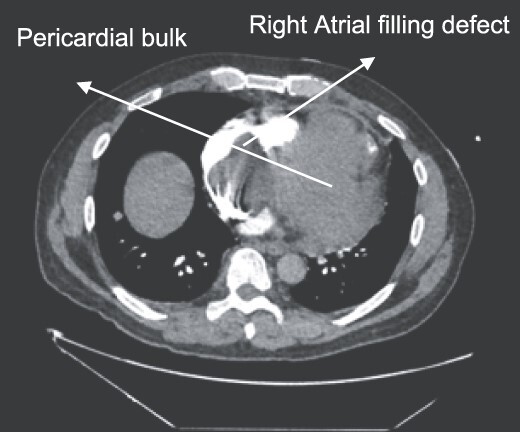
CTPA showing oval-shaped heterogenous lesion as well as RA filling defect.

**Figure 2:**
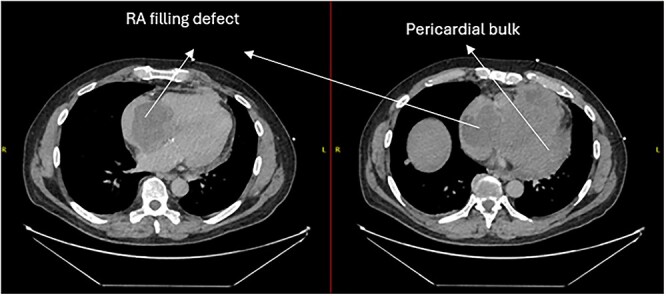
CT thorax-abdomen pelvis, depicting the RA filling defect as well as pericardial bulk.

**Figure 3:**
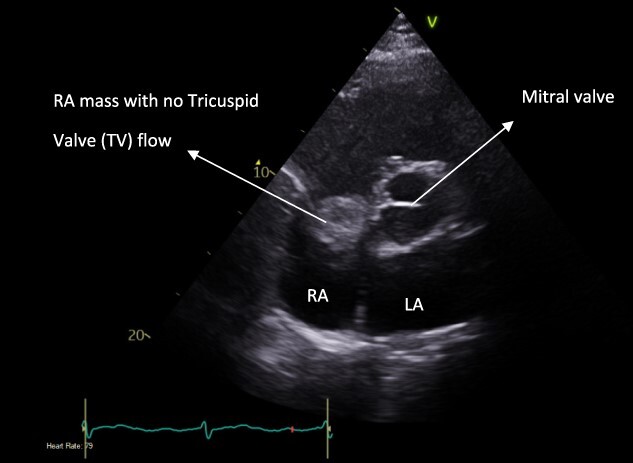
Pre-operative apical four-chamber view echocardiogram showing RA bulk blocking TV flow.

A 52-year-old gentleman was admitted to a district general hospital for shortness of breath causing type 1 respiratory failure with oxygen saturations of <90% despite high flow oxygen. His past medical history was unremarkable, and he was usually fit and well. A CT Pulmonary Angiogram (CTPA) was undertaken, which showed a heterogeneous oval-shaped lesion measuring 15 × 9 × 14 cm^3^ between the heart and the diaphragm, with a median attenuation of 35 HU and no enhancement during the arterial phase. Further solid lesions of 14 mm in the right lower lobe and 5 mm in the right upper lobe raised the suspicion of a primary cardiac sarcoma with pulmonary metastasis. This is shown in Fig. 1. Subsequent urgent CT staging (Fig. 2) was undertaken, which confirmed an indeterminate space occupying the inferior pericardial space, right atrial (RA) filling defect and anterior nodular pericardial thickening. No intra-abdominal or bone lesions were identified. An echocardiogram also confirmed these finding with the addition of no flow through the tricuspid valve (TV), as shown in Fig. 3.

Due to the critical condition of the patient, he was transferred to a tertiary centre for an emergency salvage debulking of the RA tumour via sternotomy. The goals were to improve haemodynamics, save his life and to obtain tissue histology samples.

The chest was divided with a median sternotomy and multiple pericardial adhesions were encountered on approach. Following adhesiolysis, the tumour was found to be invading the inferior surface of the diaphragm and right ventricle. This was determined to be non-resectable. Hence, the cannulation strategy was through superior vena cava, right femoral venous percutaneous cannulation and the ascending aorta. The right atrium was incised, and the tumour was debulked. Once the inferior vena cava and TV were free, the atrium was closed with 4/0 sutures. The cardiopulmonary bypass time was 43 minutes and the cross-clamp time was 15 minutes.

Tissue samples were sent for histology. Macroscopically, three pieces of grey and tan tissue measuring 100 × 80 × 15 mm^3^ were found to be heterogeneous. Microscopic findings showed a malignant hypercellular neoplasm with vague fascicular to haemoangiopericytic architecture with scant intervening stroma and amphophilic cytoplasm. There was some cytoplasmic dot staining with pancytokeratin, and patchy staining with CD56 and CD99. This confirmed a synovial sarcoma that was Grade 3 (FNCLCC) with the tumour extending to the margins.

Post-operatively, the patient required stay in intensive care for 12 days for ventilatory and inotropic support. During this period, the patient required a short course of antibiotics for a hospital-acquired pneumonia. However, he was subsequently stepped down to the ward and discharged on warfarin with a target INR of 2.5. Following the diagnosis of synovial sarcoma, the patient was referred to oncology for follow-up and chemotherapy. On a 15 months’ follow-up, the patient was still alive and undergoing adjuvant therapy, although the prognosis is still uncertain.

**Figure 4:**
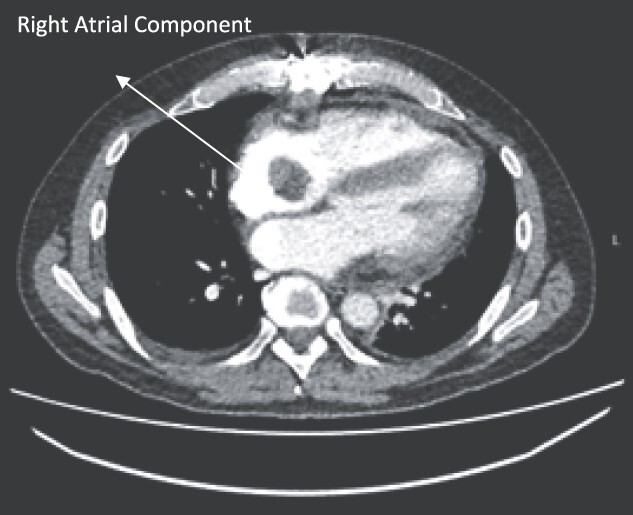
Post-operative CT scan during chemotherapy showing reduction of size RA bulk.

**Figure 5:**
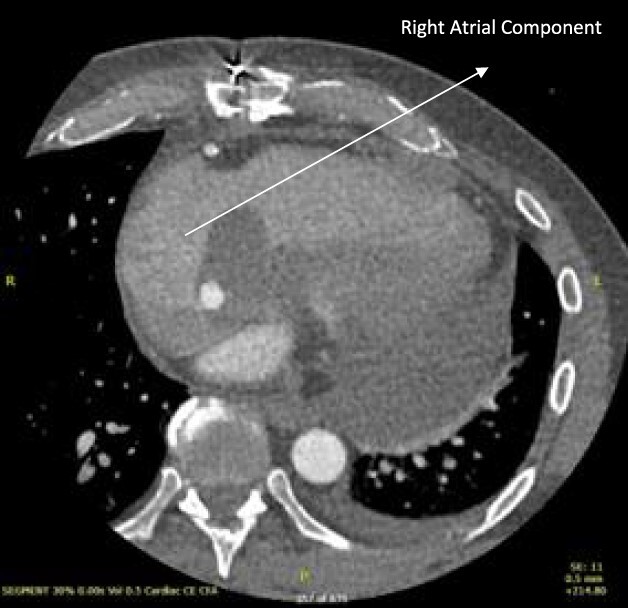
Post-operative CT scan showing further reduction of RA bulk following chemotherapy.

**Figure 6:**
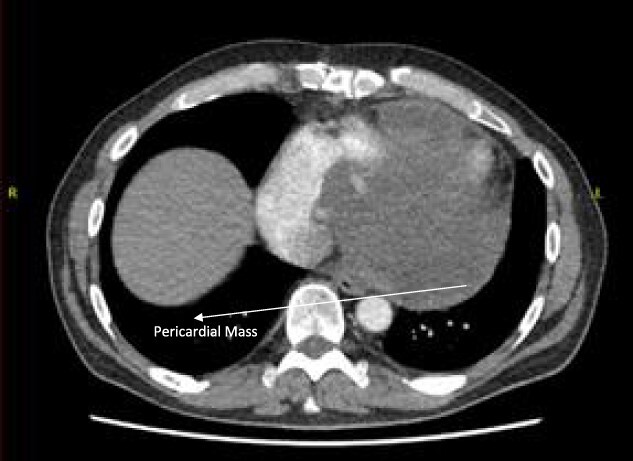
Post-operative CT scan showing pericardial mass following chemotherapy.

Follow-up CT scans were undertaken during chemotherapy that showed continued reduction in the size of the RA tumour, as demonstrated in Figs 4 and 5. The pericardial mass following chemotherapy is visualized in Fig. 6.

## Discussion

The incidence of a pericardial synovial sarcoma is rare with only a few cases being reported in literature [1, 2]. A series of 12 485 autopsies identified the incidence of 0.056% for primary cardiac tumours [3]. Pericardial tumours can be benign or malignant with an incidence of 80 and 20%, respectively [4]. Synovial sarcomas have been described to belong to three subtypes: biphasic, monophasic and poorly differentiated [2]. The relationship between immunohistochemistry markers and the variants of synovial sarcoma is not fully established; however, Bcl-2, CD99 and cytokeratin have been described as characteristic markers of synovial sarcomas [5, 6].

Factors for a good prognosis and increased survival from the limited data available were noted to be location on the left side of the heart, limited necrosis, low mitotic count, no metastasis on diagnosis. Their case series had 75 patients of which the mean and median survival were 11 and 6 months, respectively [4]. However, this patient had none of these features and was undergoing chemotherapy under oncology 15 months post-operatively. Prognosis can also be directly linked to debulking, and the quantity of the tumour resected. In fact, less than a quarter of published cases have been identified to have been completely resectable due to the anatomical location and typically aggressive spread [2].

Symptoms of pericardial synovial tumours were mostly attributed to chest pain and dyspnoea, which have been noted to be attributed to an increased pericardial effusion. However, despite the large tumour, no effusion was demonstrated on echocardiogram. This case report demonstrates the patient was that of an acute scenario and an atypical presentation. Typically, careful pre-operative planning and staging is required with the use of echocardiogram, CT imaging, MRI and cytology of any pericardial effusion. However, this case has demonstrated that a successful salvage operation is feasible with adequate debulking and prompt oncology referral. A swift response is paramount to maximize prognosis.
